# Correction: Analysis of coupling in geographic information systems based on WASPAS method for bipolar complex fuzzy linguistic Aczel-Alsina power aggregation operators

**DOI:** 10.1371/journal.pone.0332316

**Published:** 2025-09-11

**Authors:** Zeeshan Ali, Khizar Hayat, Dragan Pamucar

There are errors in the author affiliations. The correct affiliations are as follows:

Zeeshan Ali^1^, Khizar Hayat^2^, Dragan Pamucar^2^

**1** Department of Information Management, National Yunlin University of Science and Technology, Douliou, Yunlin, Taiwan, **2** Department of Mathematics, University of Kotli, AJ&K, Pakistan, **3** Sze´chenyi Istva´n University, Győr, Hungary

## References

[1] Ali Z, Hayat K, Pamucar D. Analysis of coupling in geographic information systems based on WASPAS method for bipolar complex fuzzy linguistic Aczel-Alsina power aggregation operators. PLoS One. 2024;19(9):e0309900. doi: 10.1371/journal.pone.0309900 39240959 PMC11379306

